# Cobalt and zinc columbite compounds as new anode materials for Na-ion batteries

**DOI:** 10.1039/d5ra05710h

**Published:** 2025-09-19

**Authors:** Y. Bhaskara Rao, C. André Ohlin

**Affiliations:** a Department of Chemistry, Umeå University Umeå 90187 Sweden andre.ohlin@umu.se

## Abstract

Niobium-based oxide anode materials are promising anode materials for sodium ion batteries because of their high structural stability, safety, fast energy storage kinetics, and promising capacity. In this work, niobate compounds of the columbite type, ANb_2_O_6_ (A = Co^2+^ and Zn^2+^), prepared by a facile sol–gel method, are investigated as new anode materials for sodium ion batteries. The poor intrinsic electronic conductivity of the niobate anode materials is successfully overcome by coating the active materials with carbon in order to enhance Na-ion transport and storage. High-resolution transmission electron microscopy images reveal uniform coating of the carbon layer around the active particles to prevent agglomeration in both materials. While the carbon-coated material CoNb_2_O_6_ (CNO) at a current density of 25 mA g^−1^ delivered a high reversible capacity of 295 mA h g^−1^, the carbon-coated ZnNb_2_O_6_ (ZNO) material yielded 262 mA h g^−1^. However, the ZNO material delivered a significant cycling stability at a current density of 200 mA g^−1^, which corresponds to a capacity retention of 72% after 50 cycles. The difference in their electrochemical performances is related to the structural defects, specific surface areas, charge transfer resistances during charge–discharge cycles, Na^+^-ion diffusion coefficients, and the contribution of capacitive and diffusive behaviours to the total capacity. The present work not only introduces columbite compounds as anode materials with promising electrochemical properties but also provides insights into the development of potential anode materials for sodium ion battery technology and offers the foundation for designing new electrode materials with enhanced ion-diffusion pathways.

## Abstract 2

Columbite niobate compounds, CoNb_2_O_6_ and ZnNb_2_O_6_, are introduced as new anode materials for sodium ion batteries.

## Graphical abstract

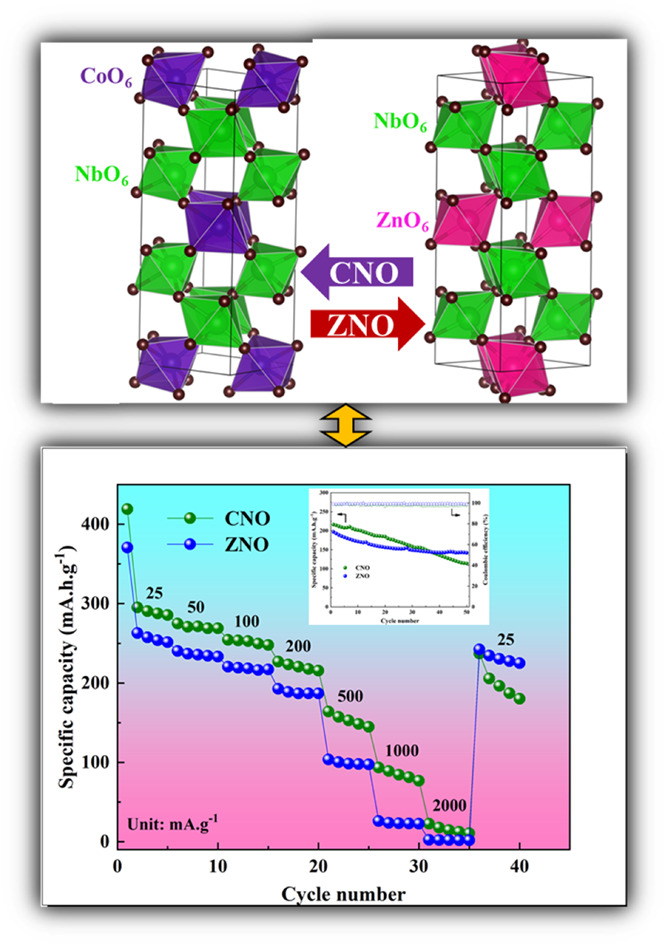

## 1. Introduction

Sodium-ion batteries (SIBs) are promising alternatives to lithium-ion batteries (LIBs) for the next-generation sustainable and large-scale electrochemical energy storage devices due to their relatively low cost and use of abundant resources.^1^ However, significant volume expansion in the electrode materials during Na^+^-ion (de)intercalation leads to poor electrochemical kinetics due to the large ionic radius of sodium (1.02 Å).^2^ Thus, SIBs suffer from poor energy storage characteristics such as low capacity and inferior rate performance compared with LIBs. Hence, it is essential to investigate new anode materials that meet the practical demands of SIBs. Metal oxides have been investigated widely as alternatives to carbon-based anodes in SIBs due to their desirable electrochemical properties.^3^ Among them, niobium based oxides attract attention because of their superior physicochemical properties and prospective application in energy-storage devices. In addition, they exhibit a high working potential (>1.0 V) that can prevent the formation of sodium dendrites, ensuring the safety of working batteries.^4^ However, application as substitutes for commercial anode materials is restricted by the low alkali ion diffusion rate in many niobium-based oxide materials that is caused by low intrinsic electrical conductivity (*ca.* 3.4 × 10^−6^ S cm^−1^ at 300 K).^5^ Several approaches have been taken to enhance the Na^+^-ion insertion/extraction kinetics, and the electronic conductivity can be greatly improved by *e.g.* selecting a favourable crystal structure/polymorph, optimizing the particle size, controlling the morphology to reduce Na^+^-ion diffusion pathways, cation or anion doping and the addition of a conductive substance, like carbon.^6^ Furthermore, because of their shorter diffusion paths, nanostructured materials with larger surface areas are expected to have better Na^+^-ion insertion or extraction kinetics, which will lead to higher power densities.^7^

Among the various methods investigated to enhance the electronic conductivity, to prevent the nanoparticles from aggregation, and to improve the SEI film, carbon coating of the active material is found to be an economically viable, facile and convenient technique.^8^ For example, core–shell structured Nb_2_O_5_@NC nanoparticles were prepared by a sonochemical approach in which polyacrylonitrile was used as a carbon and nitrogen source.^9^ The nano-composite delivered a discharge capacity of 96 mA h g^−1^ after 200 cycles at 0.2C, which is a significant improvement compared to the undoped sample (28 mA h g^−1^). Kim *et al.* reported a carbon coated Nb_2_O_5_ material with a mesoporous structure which was prepared by employing a one-pot synthesis technique with block copolymer assistance.^10^ At a current density of 50 mA g^−1^, the material, m-Nb_2_O_5_–C, exhibited a reversible capacity of *ca.* 175 mA h g^−1^. On the other hand, Chen *et al.* prepared Nb_2_O_5_/g-CNT composite by a one-step hydrothermal method followed by annealing.^11^ The composite offered a high specific capacity of 203 mA h g^−1^ at current density of 0.2 A g^−1^.

We have previously reported a fluorine-doped and carbon-coated KNb_3_O_8_ material synthesized by a solution-assisted solid-state reaction, which delivered a reversible capacity of 173 mA h g^−1^ at a current density of 10 mA g^−1^.^12^ Here, polyvinylidene fluoride (PVDF), a commonly used binder for SIBs/LIBs, is utilized as both carbon and fluorine source.

Herein, we report columbite niobate compounds, ANb_2_O_6_ (A = Co^2+^ and Zn^2+^), prepared by a facile sol–gel method, as new anode candidates for Na-ion battery applications. Further, the materials CoNb_2_O_6_ and ZnNb_2_O_6_ are coated with carbon, where citric acid is used as a carbon source, in order to enhance the electrochemical performance of the electrodes. The transport and storage behaviour of Na^+^ ions in both materials are investigated and compared with respect to their structure, disordered/defective nature, morphology, specific surface area, Na^+^-ion diffusion coefficient and the contribution of capacitive and diffusive behaviour. X-ray diffraction, scanning electron microscopy (SEM) and transmission electron microscopy (TEM and HRTEM) were used to determine crystalline structure and sample morphology. The vibrational modes, along with the D and G bands, were analyzed with the help of Raman spectroscopy. Further, the specific surface area and pore size distributions were investigated by using N_2_ adsorption/desorption isotherms. The electrochemical performance of the coin cells were tested with the help of cyclic voltammetry, galvanostatic charge–discharge cycles, Nyquist impedance plots and the total capacitance partition curves.

## 2. Experimental section

### 2.1 Material synthesis

Carbon coated-CoNb_2_O_6_ and -ZnNb_2_O_6_ materials were prepared by a facile sol–gel method. In a typical reaction, appropriate amounts of niobic acid (Nb_2_O_5_·*n*H_2_O, >99%, MW (Nb_2_O_5_): 265.81 g mol^−1^, 40% H_2_O w/w; UC Davis, 1.877 g), cobalt nitrate hexahydrate (Co(NO_3_)_2_·6H_2_O, >98%, MW: 291.03 g mol^−1^, Sigma-Aldrich; 1.708 g) or zinc nitrate hexahydrate (Zn(NO_3_)_2_·6H_2_O, >98%, MW: 297.47 g mol^−1^, Alfa-Aeser; 1.714 g), and citric acid (C_6_H_8_O_7_, >98%, MW: 192.12 g mol^−1^, TCI, 1.128 g) were mixed in 50 ml deionized water and magnetically stirred at 70 °C until the water was evaporated. The dried product was ground in an agate mortar and pestle and then calcined at 700 °C for 6 h under N_2_ atmosphere to obtain the final carbon coated-CoNb_2_O_6_ and -ZnNb_2_O_6_ powders. These carbon-coated samples are referred to as CNO and ZNO, respectively.

### 2.2 Material characterization

X-ray diffraction (XRD) spectroscopy using Cu-K_α_ radiation on a PANalytical Xpert3 powder X-ray diffractometer was used to analyze the crystal structure of the materials. The samples were scanned at a step size of 0.02626° in the 2*θ* range of 20–90°. The Raman spectra were recorded using a Renishaw Qontor device (Renishaw Plc, UK) running WiRe (version 5.3). In normal confocality mode, a 532 nm solid state laser with a nominal maximum power of 50 mW was employed through a 20× lens. A Zeiss Merlin Schottkey FEG-SEM fitted with a GEMINI II column was used to capture scanning electron microscopy (SEM) images. The working distance, the prober current, and the acceleration voltage (or electron high tension, EHT) were accompanied with the corresponding images. A Ceta-D 4k × 4k CMOS detector was used to capture high resolution transmission electron microscopy (HRTEM) images on the FEI Glacios 200 kV cryo-TEM. Copper TEM grid mesh (400 squares per inch) was applied to a lacey carbon film to obtain the TEM grids. For selected area electron diffraction (SAED) pictures, an aperture of 40 μm or 100 μm was used to remove anything other than the particle of interest. Measurements of N_2_ adsorption–desorption were performed with a Micromeritics TriStar 3000 porosimeter. After the samples were outgassed for two hours at 120 °C, the isotherms were taken at −196 °C. The Brunauer–Emmett–Teller (BET) method was used to gather the specific surface areas, and the desorption isotherms were used to compute the pore volumes. The ASAP-2010 software and the Barret, Joyner, and Halenda (BJH) algorithm were used to estimate the pore size distributions.

### 2.3 Electrochemical characterization

CR-2032 coin-type half-cells were prepared for the electrochemical experiments in an Ar-filled glove box (Mbraun, MB10 compact) with O_2_ and H_2_O concentrations <0.5 ppm. To prepare a slurry, deionized water was combined with the active material (80 wt%), Super P (Thermo Scientific) (10 wt%), and sodium carboxymethylcellulose (CMC-MedChemExpress; 10 wt%) binder. The mixture was applied on a copper foil current collector (TMAXCN; 14 mm in diameter and 0.1 mm in thickness) and dried at 50 °C. The average mass loading of electrode was 2–3 mg cm^−2^. The glass microfibre filter (Whatman, Grade GF/D; 19 mm diameter) was employed as the separator, and the liquid electrolyte was 1 M NaClO_4_ (Thermo Scientific) in ethylene carbonate (EC, from AmBeed) and dimethyl carbonate (DMC, from TCI; 1 : 1 v/v). Na metal was used as the counter/reference electrode. Electrochemical impedance spectroscopy (EIS) in the frequency range of 10 mHz to 1 MHz and the cyclic voltammetry (CV) curves (0.01–2.5 V) at various scan rates were recorded on a Gamry 1010E interface workstation. A NEWARE CT-4008 battery tester was used to record the galvanostatic charge–discharge curves within a voltage window of 0.01 to 2.5 V (*vs.* Na^+^/Na). All electrochemical measurements were carried out at 20 °C.

## 3. Results and discussion

**Fig. 1:**
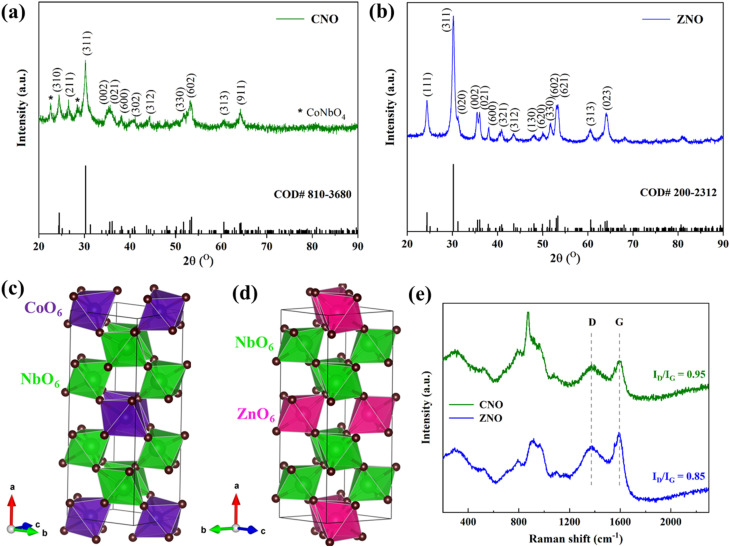
XRD patterns (a and b), crystal structure (c and d) of CNO and ZNO materials, respectively, and (e) Raman spectra of CNO and ZNO materials.

The columbite compounds CNO and ZNO were prepared by a simple sol–gel process at a low calcining temperature of 700 °C. The XRD patterns of the materials, CNO and ZNO are shown in Fig. 1a and b. All diffraction peaks of the sample, CNO are indexed to orthorhombic-CoNb_2_O_6_ (COD# 810-3680) with a *Pbcn* space group,^13^ except a minor monoclinic-CoNbO_4_ (COD# 200-2264) phase. On the other hand, a highly pure orthorhombic phase is obtained in the ZNO sample (COD# 200-2312) with a *Pbcn* space group matching all diffraction peaks, without any secondary phase.^5^ The corresponding (*hkl*) indices are represented for both samples after refining the XRD patterns by using the Rietveld fit. The observed and calculated XRD patterns of ZNO sample are displayed in Fig. S1 and the lattice parameters of CNO and ZNO materials are given in Table S1. The values are in good agreement with those reported in the literature.^5,13^ The unit cell volume is found to be slightly higher in case of ZNO sample. Further, the *d*-spacing values for (311) crystal planes are nearly equal and they are 0.2948 and 0.2956 nm for CNO and ZNO samples, respectively. The average crystallite size (*D*) can be determined by using Debye–Scherrer equation,1
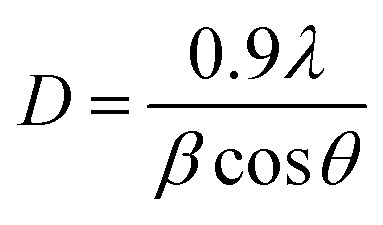
where, *λ* is the wavelength of X-rays used (1.54 Å), *β* is full width at half maximum (FWHM) of the diffraction peak (0.00842 rad for CNO and 0.007159 rad for ZNO) and *θ* is the Bragg angle of the diffraction peak (15.1524° for CNO and 15.1087° for ZNO). After substituting all the values, the average crystallite size values are found to be 17.05 and 20.05 nm for CNO and ZNO samples, respectively. In a unit cell, there are eight NbO_6_ and four AO_6_ (A = Co^2+^ or Zn^2+^) corner-sharing octahedra, connected by the oxygen atoms, as illustrated in Fig. 1c and d. The ANb_2_O_6_ structure exhibits three different oxygen sites, which are surrounded by different numbers of A^2+^ and Nb^5+^ ions.^13^ Further, the vibrational energy modes as well as the degree of disorder in both carbon-coated samples of CNO and ZNO were analyzed using Raman spectroscopy (Fig. 1e). The peaks observed below 380 cm^−1^ in the spectra belong to bending and torsion modes of O–Nb–O in NbO_6_ octahedra. The modes that correspond to stretching movements inside the NbO_6_ octahedra are located in the range of 380–800 cm^−1^.^14^ The Raman vibrational mode observed in the vicinity of 520 cm^−1^ in both samples is mainly corresponding to the bridging Nb-O bond stretching vibration.^15^ There is a noticeable sharp peak at 873 cm^−1^, which could be caused by the vibrational mode corresponding to a strongly distorted unbridged [NbO_6_] octahedra. The broad bands located within the range of 500–1000 cm^−1^ correspond to slightly distorted unbridged [NbO_6_] octahedra in both samples.^15^ Furthermore, the D band to G band intensity ratio (*I*_D_/*I*_G_) is a parameter used in Raman spectroscopy (Fig. 1e) to assess the level of disorder and defects present in carbon-based materials. A direct proportionality between the *I*_D_/*I*_G_ ratio and structural defects is observed by Moon *et al.*^16^ and the structural defects can improve the ion and electron accessibility by increasing the surface area and porosity of carbon materials.^17^ Here, the D band *ca.* 1380 cm^−1^ arises from the disorder-induced vibrations, indicating the presence of defects, and the G band *ca.* 1600 cm^−1^ is the characteristic of the graphitic structure. Table 1 makes it evident that both materials have an *I*_D_/*I*_G_ ratio below 1, which suggests that there is more sp^2^-bonded graphitic carbon than sp^3^-bonded disordered carbon. In particular, the material, CNO yielded a higher *I*_D_/*I*_G_ ratio (0.95) compared to that of the material, ZNO (0.85) indicating a greater degree of disorder or defectiveness in CNO. Thus, the results show that there is a comparatively higher percentage of amorphous carbon than crystalline carbon, and the synthesized samples will have a high level of electronic conductivity since amorphous carbon is an electronic conductor.^18^

**Table 1:** Comparison of crystallite size, *I*_D_/*I*_G_ ratio, specific surface area (*S*_BET_), reversible discharge capacity at 25 mA g^−1^, capacity retention at 200 mA g^−1^ after 50 cycles, charge transfer resistance (*R*_ct_) after 10 cycles and diffusion coefficient for CNO and ZNO samples

Material	Crystallite size *D* (nm)	*I*_D_/*I*_G_	*S*_BET_ (m^2^ g^−1^)	Reversible capacity at 25 mA g^−1^ (mA h g^−1^)	Capacity retention (%)	*R*_ct_ (Ω)	Diffusion coefficient (cm^2^ s^−1^)
CNO	17.05	0.95	30.58	295	53	44.6	7.6 × 10^−12^
ZNO	20.05	0.85	37.44	262	72	28.1	9.3 × 10^−12^

**Fig. 2:**
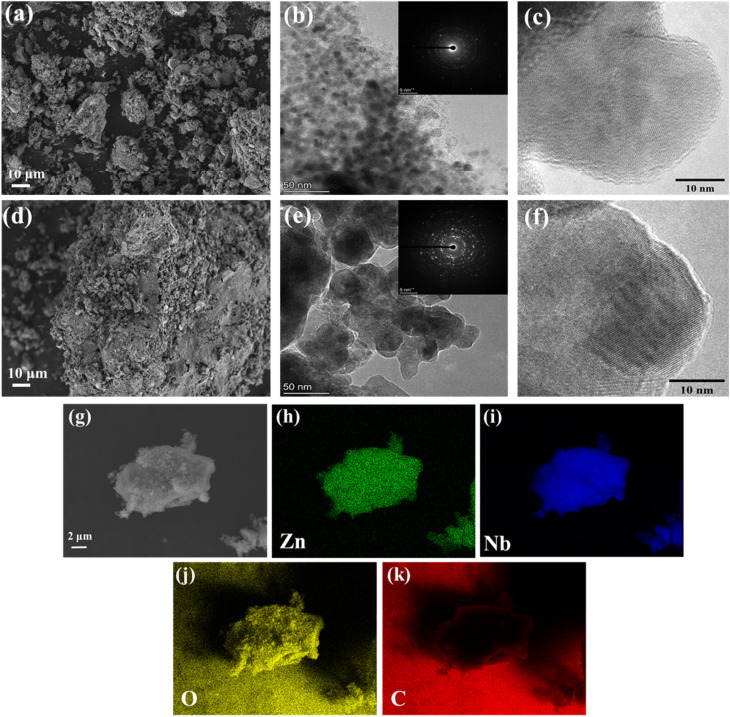
SEM (a and d), TEM (b and e) and HRTEM (c and f) images of (a–c) CNO, and (d–f) ZNO materials (inset: SAED pattern), and (g–k) electron diffraction X-ray spectroscopy (EDX) images of ZNO material.

The morphology and microstructure of the CNO and ZNO materials were analyzed by SEM and TEM (Fig. 2). From Fig. 2a and d it is clear that both samples exhibit micron-sized particles of irregular shape and size. The TEM images (Fig. 2b and e) reveal the morphology of both carbon coated samples and the crystal orientations of CNO (inset: Fig. 2b) and ZNO (inset: Fig. 2e) materials are evident from the selected area electron diffraction (SAED) pattern. Specifically, the hollow ring pattern along with the bright spots seen in the images indicate that an amorphous or disordered carbon framework envelops the active particles.^19,20^ The lattice fringes in both materials are further visible in the HRTEM images (Fig. 2c and f), indicating their crystalline nature. The enlarged view (shown in Fig. S2a and b) of HRTEM images reveals that the *d*-spacing values of 0.36 and 0.37 nm correspond to the (310) and (111) planes of CNO and ZNO materials, respectively. These values are in good agreement with those previously reported,^5,13^ moreover, the SAED patterns from Fig. S2c and d illustrate a few planes which are well consistent with the XRD data shown in Fig. 1a and b. Thus, the columbite anode compounds are successfully incorporated and decorated with the carbon matrix, as seen by the thin amorphous layer surrounding the active particles in the HRTEM images. Finally, the EDX elemental mapping images in Fig. 2h–k confirm the uniform distribution of Zn, Nb, O and C in the carbon-coated material, ZNO.

**Fig. 3:**
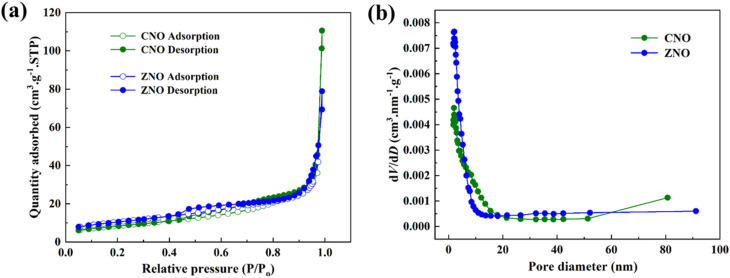
(a) N_2_-adsorption and desorption isotherms, and (b) pore size distribution of CNO and ZNO materials.

N_2_ adsorption–desorption isotherms were obtained in order to determine the surface area and porosity of the synthesized CNO and ZNO samples (Fig. 3a and b) and these adsorption–desorption isotherms show a type-III behaviour which indicates that the interaction between the adsorbent and adsorbate are weak.^5,21^ The ZNO-material exhibited a specific surface area of 37.44 m^2^ g^−1^ and a micropore volume of 0.00057 cm^3^ g^−1^, whereas, CNO exhibited a specific surface area of 30.58 m^2^ g^−1^ and a micropore volume of 0.00026 cm^3^ g^−1^. It was earlier reported that the incorporation of carbon matrix can suppress the crystal growth of bulk material and produce samples with a large surface area.^22^ Thus, the large surface area offers more number of active sites for ion adsorption and provides high potential for the formation of aligned pore channels which enable an easy and open transportation of electrolyte ions.^23^ Further, Fig. 3b reveals that the CNO and ZNO samples have Barrett–Joyner–Halenda (BJH) mean pore diameters of 22 and 13 nm, respectively. Thus, the structure with a large surface area can buffer volume change, reduce the transport distance for ion diffusion, as well as stress evolution generated during the ion insertion/extraction process.^23^

**Fig. 4:**
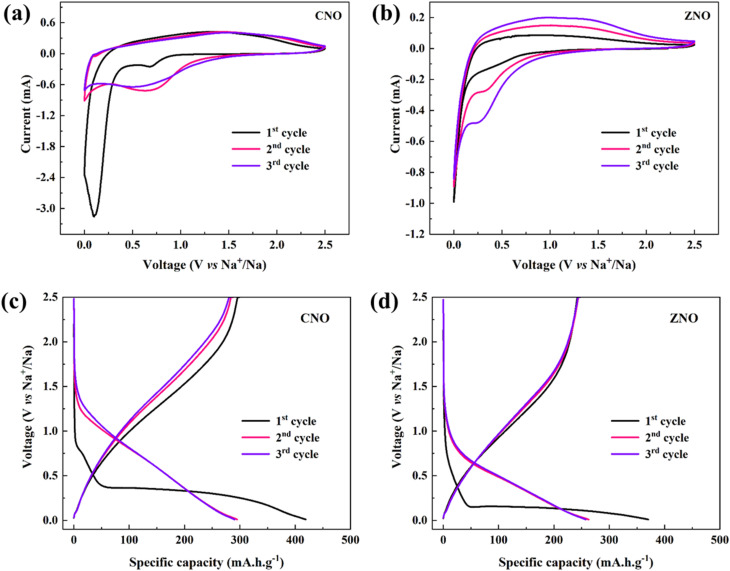
Cyclic voltammetry of (a) CNO, and (b) ZNO materials, and galvanostatic charge–discharge curves of (c) CNO, and (d) ZNO materials for the first three cycles.

The cyclic voltammogram curves for the CNO and ZNO electrodes for the first three cycles, with a scan rate of 0.2 mV s^−1^, in the voltage range of 0.01 to 2.5 V are displayed in Fig. 4a and b. There are two reduction peaks in the first CV curve at 0.69 V and 0.1 V that can be found in the CNO sample. These peaks are attributed to the Na^+^-ion intercalation and the formation of SEI layer, respectively.^24^ In ZNO electrode, the broad reduction peak appear at *ca.* 0.3 V indicates the generation of irreversible SEI layer after Na^+^-ion insertion reaction.^25^ The oxidation/reduction peaks in both materials become more symmetrical in subsequent cycles and are highly reversible. Thus, sodiation in the CNO and ZNO materials proceeds primarily through an intercalation mechanism.

Further, the electrochemical performance of both samples in the initial three charge–discharge cycles at a current density of 25 mA g^−1^ is shown in the Fig. 4c and d. The CNO electrode delivered initial discharge and charge specific capacities of 419.10 and 299.25 mA h g^−1^, respectively, whereas ZNO delivered initial discharge/charge capacities of 370.56 and 245.78 mA h g^−1^, respectively, which correspond to initial coulombic efficiencies (ICE) of 71% and 66% for the two materials. The lower ICE is recorded by the ZNO material due to its larger specific surface area which ultimately affects the formation of SEI layer during the first cycle. The high specific surface area and micropores may absorb more alkali ions, forming “dead Na”, thus increasing the irreversible capacity, which leads to a low ICE in the ZNO material.^26^ However, both materials recorded high coulombic efficiencies of over than 95% in subsequent cycles and thus exhibited high reversible electrochemical performance.

**Fig. 5:**
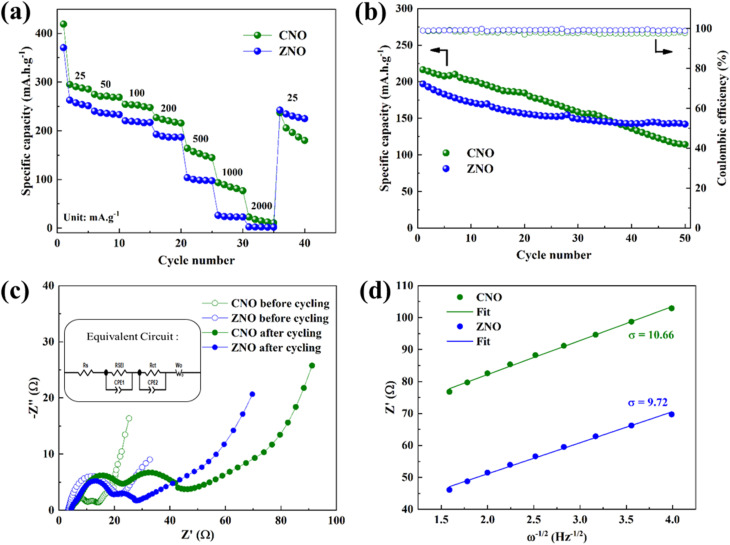
(a) Rate performance at different current densities, (b) cycling stability at a current density of 200 mA g^−1^, (c) Nyquist plots, before and after cycling (inset: equivalent circuit), (d) *Z*′ and *ω*^−1/2^ fitting curves at a low frequency region of CNO and ZNO samples.

The rate performances of both CNO and ZNO electrodes at different current densities are shown in Fig. 5a. The CNO sample displayed high discharge specific capacities of 295, 274, 254, 226, 164, 93, and 22 mA h g^−1^ at current densities of 25, 50, 100, 200, 500, 1000, and 2000 mA g^−1^, respectively, while ZNO delivered discharge specific capacities of 262, 240, 220, 192, 103, 25, and 2 mA h g^−1^, respectively, at the same current densities. Here, CNO delivered higher specific capacities compared to ZNO which may be due to the presence of more structural defects (Fig. 1e) in the CNO sample. However, good reversibility and stability are observed in the ZNO sample when reverting to the initial current density of 25 mA g^−1^. The structural integrity is better preserved in the ZNO sample compared to CNO which is reflected in the long-term cycling performance. Fig. 5b shows the electrochemical performance of CNO and ZNO samples for 50 cycles at a current density of 200 mA g^−1^. Here, ZNO delivered a high discharge specific capacity of 142 mA h g^−1^ after 50 cycles which corresponds to a significant capacity retention of 72%. Thus, increasing the specific surface area of the active materials speeds up the diffusion of mobile ions and enhances the electrochemical performance of the sample.^22^ On the other hand, CNO delivered a discharge specific capacity of only 114 mA h g^−1^ (53%) after 50 cycles. Both materials exhibited *ca.* 99% coulombic efficiencies. Thus, during cycling, the high specific surface area and large pore volume of the ZNO material facilitates the Na^+^-ion transport and suppress the volume change by preserving its structure.

The stable and high performance in the ZNO sample can be well understood by analysing the electrochemical impedance spectroscopy which is shown in Fig. 5c. The Nyquist plots, for both samples, before and after 10 cycles, are fitted using the equivalent circuit (inset of Fig. 5c). It consists of the solution resistance *R*_s_, SEI layer resistance *R*_SEI_, and the charge transfer resistance *R*_ct_. The charge transfer resistances of 5.9 and 16.7 Ω are recorded for CNO and ZNO samples, respectively, before cycling. However, a larger charge transfer resistance of 44.6 Ω is observed for the CNO sample compared to that of the ZNO sample (28.1 Ω), after cycling. Thus, the CNO sample exhibited a high resistance during cycling which may be influencing the long-term cycling stability.

Additionally, the Na^+^-ion diffusion coefficient, *D*_Na^+^_, may be found using eqn (2),^27^ and is directly related to the charge transfer resistance and enhanced electrochemical stability.2
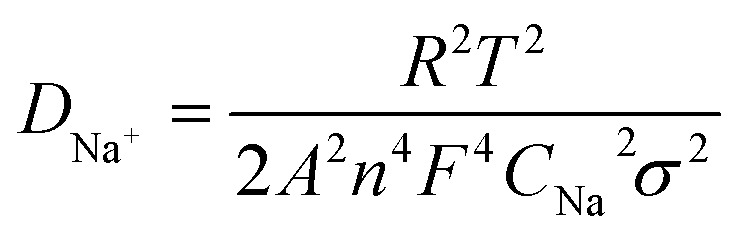
where *R*, the universal gas constant (8.314 J mol^−1^ K^−1^), *T*, temperature (293 K), *F*, Faraday constant (96 485 C mol^−1^), *n*, number of transferred electrons (1), *A*, effective working area of the electrode (1.5386 cm^2^), *σ*, the slope of *Z*′ *vs. ω*^−1/2^ (9.72 for ZNO and 10.66 for CNO) and *C* represents the Na^+^-ion concentration (4.048 × 10^−3^ mol cm^−3^ for ZNO and 4.082 × 10^−3^ mol cm^−3^ for CNO). Using eqn (3),

3 *Z*′ = *R*_s_ + *R*_SEI_ + *R*_ct_ + *σω*^−1/2^

The slope (*σ*) can be calculated by using the plot between *Z*′ and *ω*^−1/2^ (Fig. 5d). Here, *Z*′ is the real part of impedance, *σ* is the Warburg factor, and *ω* is the angular frequency.^28^ After substituting all the values, the ZNO electrode exhibited a sodium ion diffusion coefficient of 9.3 × 10^−12^ cm^2^ s^−1^, whereas CNO achieved 7.6 × 10^−12^ cm^2^ s^−1^. Thus, the transport of Na^+^-ions is somewhat more rapid in the case of ZNO, which may be due to the wider spread of liquid electrolyte over the larger surface area. Table 1 shows the comparison of various parameters along with the electrochemical performance of both CNO and ZNO materials.

**Fig. 6:**
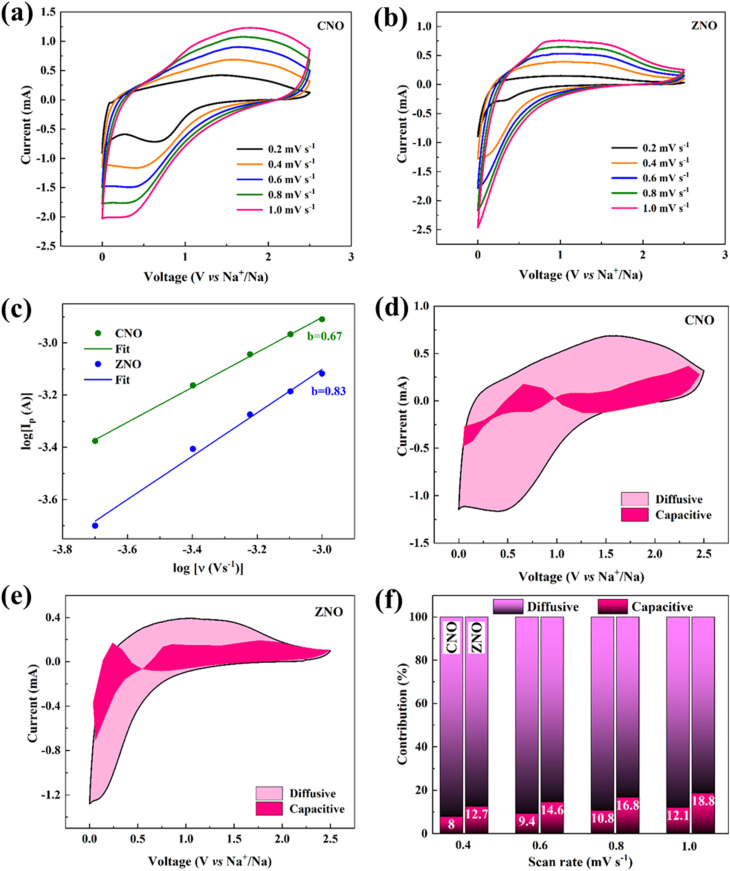
Cyclic voltammogram at different scan rates for (a) CNO and (b) ZNO materials. (c) Plot of log(ν) *vs.* log(*i*_p_) of CNO and ZNO materials. CV curve displaying the diffusive and capacitive contribution of (d) CNO, and (e) ZNO materials at 0.4 mV s^−1^ scan rate, and (f) percentage contribution of diffusive and capacitive processes at different scan rates for CNO and ZNO samples.

Fig. 6a and b display the CV plots of the CNO and ZNO materials at different scan rates. The anodic and cathodic peak currents for both materials rise with increasing scan rate, and the improved reversibility in the electrodes is indicated by their better symmetry in the redox peaks. Furthermore, a slight increase of the positions of the oxidation peaks towards positive potential suggests that there is a less polarization in both electrode materials.^29^ The power law relationship is used to better investigate the electrode kinetics in both materials. By using eqn (4),^30^

4 *i*_p_ = *aν*^*b*^

where *i*_p_, the peak current, *ν*, the scan rate, and *a* and *b* are constants determined from fitting. Eqn (4) can be modified as eqn (5).

5 log(*i*_p_) = log(*a*) + *b* log(*ν*)

A linear fit of the plot between log(*ν*) and log(*i*_p_) (Fig. 6c) can be used to determine the slope *b*, which can then be used to evaluate the diffusion-controlled and capacitive contributions. From Fig. 6c, the *b* values of CNO and ZNO materials are found to be 0.67 and 0.83, respectively, demonstrating that both the capacitive and diffusive processes regulate the Na^+^-ion storage mechanism. Furthermore, eqn (6) (ref. 31) provides the quantitative repartition of the capacitive and diffusive contributions to the overall storage mechanism in both materials.

6 *i*(*V*) = *k*_1_*ν* + *k*_2_*ν*^1/2^

where, *k*_1_*ν* and *k*_2_*ν*^1/2^ stand for the surface capacitive and the diffusion-controlled contributions, respectively. Fig. 6d and e display the capacitive and diffusive contributions to the CV plots of the CNO and ZNO electrodes at a scan rate of 0.4 mV s^−1^. Fig. 6f makes it evident that as the scan rate rises from 0.4 to 1 mV s^−1^ in both materials, the percentage of capacitive contribution progressively increases. This is due to the fact that surface capacitance dominates the total capacity at higher rate conditions. At the same time, the diffusion-regulated capacity contribution decreases because the slow intercalation speed is unable to satisfy the needs of the high rate of electrochemical processes at higher scan rates.^32^ Thus, the ZNO electrode exhibited better electrochemical performance due to its high specific surface area, large pore volume and high Na^+^-ion diffusion coefficient.

## 4. Conclusion

The orthorhombic columbite compounds ANb_2_O_6_ (A = Co^2+^ and Zn^2+^) were prepared by a facile sol–gel method followed by low temperature (700 °C) annealing. Further, the active particles were successfully embedded in a carbon matrix to improve the Na^+^-ion storage and transport capability. As a result, the carbon-coated cobalt-containing sample CNO achieved a higher rate performance (164 mA h g^−1^ at 500 mA g^−1^) at every current density compared to that of the zinc-based ZNO material (103 mA h g^−1^) due to the improved structural defects. Conversely, the ZNO sample delivered a good cycling stability at 200 mA g^−1^ corresponding to a capacity retention of 72% over 50 cycles. This type of differing electrochemical behaviour between the CNO and ZNO materials is explained in terms of their structural defects, specific surface area, pore volume, Na^+^-ion diffusion coefficient, charge transfer resistances, and inherent pseudocapacitive behaviour. Thus, the present study highlights columbite compounds as new and promising anode candidates for the development of high performance sodium ion battery technology.

## Author contributions

Y. Bhaskara Rao: investigation, methodology, conceptualization, formal analysis, data curation, writing – original draft. C. André Ohlin: validation, supervision, resources, visualization, funding acquisition, writing – review & editing, project administration.

## Conflicts of interest

The authors have no conflicts of interest to declare.

## Supplementary Material

RA-015-D5RA05710H-s001

## Data Availability

Data supporting this study is included within the article; additional data is included as SI. See DOI: https://doi.org/10.1039/d5ra05710h.
