# Supplementary material for: Cobalt and zinc columbite compounds as new anode materials for Na-ion batteries
Source: RSC Adv. 2025 Sep 19;15(41):34300–9. doi: 10.1039/d5ra05710h (PMC12447704; doi:10.1039/d5ra05710h)
Supplement: RA-015-D5RA05710H-s001 [file RA-015-D5RA05710H-s001.pdf]

Supplementary Information (SI) for RSC Advances.
This journal is © The Royal Society of Chemistry 2025

Supplementary Information

Cobalt and zinc columbite compounds as new anode materials for Na-ion batteries

Y Bhaskara Rao, C. André Ohlin*

Department of Chemistry, Umeå University, Umeå 90187, Sweden

***Corresponding author: andre.ohlin@umu.se**

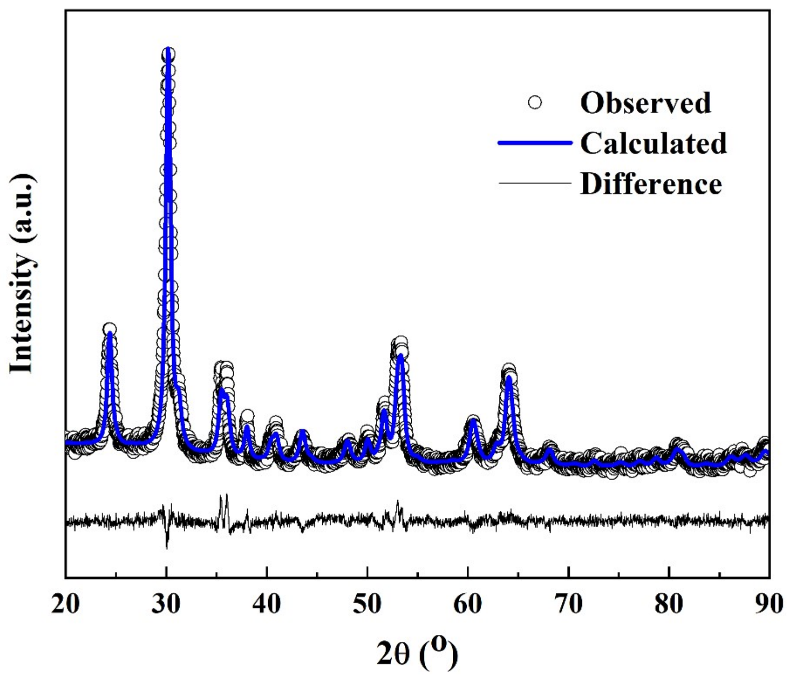

Fig. S1. Rietveld refinement of the XRD patterns of ZNO sample.

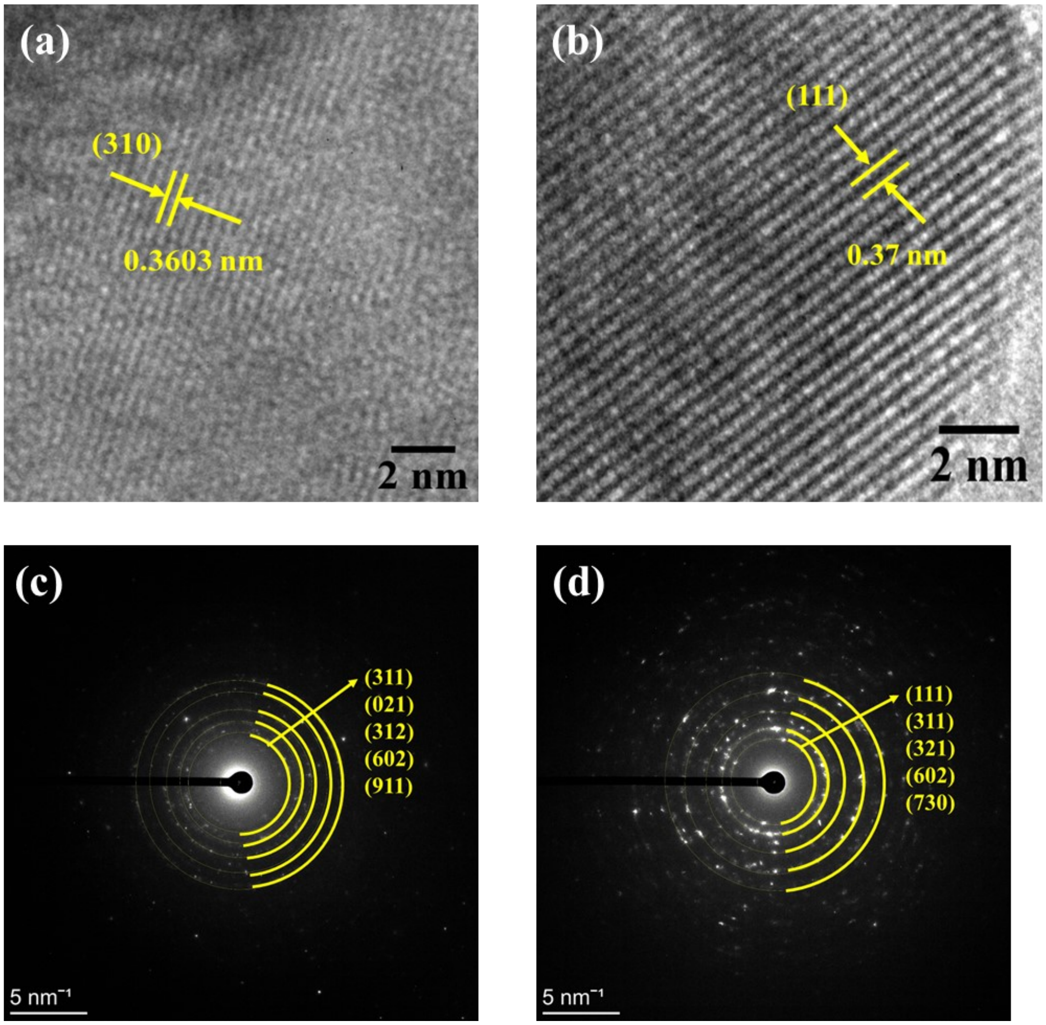

Fig. S2. Enlarged view of HRTEM images of **a)** CNO and **b)** ZNO materials, and SAED patterns of **c)** CNO and **d)** ZNO materials.

Table S1. Structural details of CNO and ZNO materials obtained from the Rietveld refinement.

Sample name	Lattice parameters			Unit cell volume (Å^3)	Goodness of fit (GoF)
	a (Å)	*b* (Å)	*c* (Å)		
CNO	14.119(8)	5.707(4)	5.048(5)	406.753(3)	1.36
ZNO	14.184(2)	5.720(1)	5.055(1)	410.124(6)	1.38
